# Effect of egg yolk of free-range chicken and methanol as a cryoprotective agent for the sperm preservation of cyprinid fish, *Neolissochilus soroides* (Valenciennes, 1842)

**DOI:** 10.1016/j.heliyon.2021.e08158

**Published:** 2021-10-11

**Authors:** Abinawanto Abinawanto, Nia Vardini, Anang Hari Kristanto, Retno Lestari, Anom Bowolaksono

**Affiliations:** a Cellular and Molecular Mechanisms in Biological System (CEMBIOS) Research Group, Department of Biology, Faculty of Mathematics and Natural Sciences, Universitas Indonesia, Depok 16424, Indonesia; b Research Institute for Freshwater Aquaculture and Fisheries Extension, Jalan Sempur No 1, Bogor 16129, West Java, Indonesia; c Graduate School of Biology, Department of Biology, Faculty of Mathematics and Natural Sciences, Universitas Indonesia, Depok 16424, Indonesia

**Keywords:** Cryoprotectants, Fertility rate, Godfish, Spermatozoa

## Abstract

The objective of this study was to determine the optimum concentration of egg yolk of free-range chicken as a cryoprotective agent on cyprinid fish, *Neolissochilus soroides* sperm after 48 h frozen. One level of methanol (10%) combined with six levels of egg yolk solution (0%, 5%, 10%, 15%, 20% and 25%) were tested. Fish Ringer's solution was used as an extender. The diluted sperm was equilibrated for 10 min at 5 °C, then kept at -10 °C temperature for 48 h. Sperm was thawed for 1 min at 40 °C. Spermatozoa viability, abnormality, and fertilization rates were analysed afterwards. The one-way ANOVA showed that the combination methanol with several concentrations of egg yolk solution had a significant effect on spermatozoa viability, abnormality, and fertilization rates (*P* < 0.05) by improving semen character. The study revealed that the 5% egg yolk solution combined with 10% methanol resulted in the highest rates of viability (82.13 ± 1.75%) and fertility rates (92.96 ± 1.94%), with the lowest abnormality (25.25 ± 2.22%). A 5% egg yolk solution was identified as the best cryoprotective agent for *N. soroides* spermatozoa preservation at -10 °C for 48 h.

## 1 Introduction

God's fish or Kancra, *Neolissochilus soroides* (Valenciennes, 1842) is one of domesticated fresh water cyprinid fish (Cypriniformes) widely distributed in Southeast Asia including Indonesia (Kottelat et al., 1996). This species also popularly known as Kancra fish in Sumedang and Kuningan areas (West Java), while in North Sumatra it is called as Batak fish (Asih et al., 2008). The populations of *N. soroides* in the wild has been decreasing due to the spawning difficulties (Junior et al., 2005), and overfishing (Rumondang and Mahari, 2017) such has been reported in the previous study in another fresh water fish (Hossain et al., 2015). Another factors like water pollution (Kottelat et al., 1996), habitat degradation, and associated, and illegal loging are also threatening the fish populations (Afrose and Ahmed, 2016; Gupta et al., 2015; Hossain et al., 2015). Therefore, the fish supply has been swift from wild populations to aquaculture productions to meet the market demand (FAO, 2020). As the bioecology of the fish in general has been well documented (Tan and Kottelat, 2009), along with the successfully developed breeding technologies (Legendre et al., 2012) the aquaculture productions are playing a key role in many emerging economies. Spawning difficulties of God's fish appeared due to the asynchronous gonad maturation (Junior et al., 2005) of the fish. The male gonad maturation of God's fish usually happens during the months of May to June, while female's from December to January (Subagja et al., 2006). Therefore, sperm cryopreservation is one of the potential solutions to overcome the asynchronous gonad maturation (Jang et al., 2017; Martínez-Páramoa et al., 2017; Riesco et al., 2017; Hezavehei et al., 2018). Cryopreservation is a cell-storage technique that maintains very low temperatures to maintain cell structure over a long period of time (Tsai and Lin, 2012). The fish sperm of many species have been cryopreserved successfully e.g. Eurasian perch, *Perca fluviatilis* (Bernáth et al., 2016); carp, *Cyprinus carpio* (Boryshpolets et al., 2017); Atlantic salmon, *Salmo salar* (Figueroa et al., 2018); zebra fish, *Dana rerio* (Matthews et al., 2018; Rebocho, 2018); Mozambique tilapia, *Oreochromis mossambicus* (Peters, 1852; Ugwu et al., 2018); rainbow trout *Oncorhynchus mykiss* (Walbaum, 1792, Ciereszko et al., 2014; Kutluyer et al., 2014); bagrid catfish, *Mystus nemurus* (Valenciennes, 1840; Muchlisin and Siti-Azizah, 2009); African catfish, *Clarias gariepinus* Burchell, 1822 (Muchlisin et al., 2015; Olanrewaju et al., 2015); giant gourami, *Osphronemus goramy* Lacépède, 1801 (Abinawanto et al., 2017); Java barb, *Barbonymus gonionotus* (Bleeker, 1850; Abinawanto et al., 2016), and sharkminnow *Osteochilus vittatus* (Valenciennes, 1842; Muthmainnah et al., 2018). However, the preservation of God's fish sperm has not been reported yet.

Cryoprotectant is a successful substance plays a vital role in preserving spermatozoa or cryopreservation of fish sperm from cold and heat shocks (Muchlisin, 2005; Agarwal, 2011; Anil et al., 2011; Chew and Zulkafli, 2012; Tsai and Lin 2012; Ciereszko et al., 2014; Muchlisin et al., 2015; Boryshpolets et al., 2017; Gil et al., 2017). However, non-natural cryoprotectants at high concentration, can be toxic to spermatozoa (Muchlisin and Siti-Azizah, 2009; Tsai and Lin, 2012; Anil, 2013; Best, 2015; Muchlisin et al., 2015; Sieme et al., 2016). Hence, it is essential to select a natural and non-toxic cryoprotectant in application for cryopreservation (Dash et al., 2008; Anil et al., 2011; Szurek and Eroglu, 2011; Tsai and Lin, 2012; Muchlisin et al., 2015). Cryoprotectant consists of two types based on the ability of penetrating the cell membrane: intracellular (permeating) and extracellular (non-permeating) and combining both types of cryoprotectant using simultaneously have been given the best results for an example, sucrose and methanol used for Indonesian giant gourami by Abinawanto et al. (2012a), skimmed milk and methanol for Java barbs by Abinawanto et al. (2012b, 2016), and honey solution, and Dimethyl Sulfoxide (DMSO) for giant gourami and shark minnow by Abinawanto et al. (2017) and Sunarma et al. (2007).

In this study we tested the combination of egg yolk of free-range chicken (layers) as a natural extracellular cryoprotectant, and methanol as a intracellular cryoprotectants with fish Ringer's solutions as an extender. Previous studies suggested the combination egg yolk and methanol have been successful in preserving the spermatozoa of Java barb (Abinawanto et al., 2013), tiger botia, *Chromobotia macracanthus* (Bleeker, 1852; Abinawanto et al., 2018) and depik, *Rasbora tawarensis* (Weber and de Beaufort, 1916; Muthmainnah et al., 2018). Therefore, the main objective of the present study is to determine the best concentration of egg yolk of free-range chicken solution combined with 10% methanol for God's fish spermatozoa storage at −10 °C for 48 h. The hypothesis of this study was there is an effect of free-range chicken egg yolk on spermatozoa quality of *Tor soro* 48 h after freezig. Further, 5% of free-range egg yolk concentration combined with 10% methanol showed the highest viability rate and fertility rate, with the lowest abnormality rate.

## 2 Material and methods

### 2.1 Time, location and broodfish preparation

The study was conducted from April to December 2019, at the hatchery of Research Installation for Freshwater Fish Genetic Resources, Cijeruk, West Java Province, Indonesia. Thirty mature males of God's fish with each body weight of 0.7–1.0 kg were cultured in a fish pond of 1.08 m long, 5.20 m wide, and 0.8 m high. They were fed on a commercial diet which has 28–30% protein content. The feed was given at rate of 2–3% of body weight two times per day (0830 h and 1630 h). Six experimental groups were assigned for four replicates in a completely randomised design.

### 2.2 Ethical approval

Health Research Ethics Committee, Faculty of Medicine, Universitas Indonesia, Cipto Mangunkusumo Hospital approved the study. Ethical approval number: KET919/UN2.F1/ETIK/PPM.00.02/2019.

### 2.3 Preparation extender and cryoprotectant

The fish Ringer's solution and egg yolk of free-range chicken (layers) were used as extender and cryoprotectant, respectively. A stock of fish Ringer's solution was prepared by dissolving 3.25 g of NaCl; 0.125 g of KCl; 0.175 g of CaCl_2_.2H_2_O; and 0.1 g of NaHCO3 in distilled water up to 500 mL, and then the solution was kept at 4 °C temperature following Abinawanto et al. (2018). The eggs of free-range chickens were purchased from local market and six different concentrations of egg yolk solution were tested: 0%, 5%, 10%, 15%, 20%, and 25%,. Following Abinawanto et al. (2018), the respective volume of egg yolk of 0, 25, 50, 75, 100, and 125 μL were added into fish Ringer's solution up to 450 μL.

### 2.4 Preparation of activator and eosin-Y solutions

The activator solution was prepared according to Perchec et al. (1995), while eosin-Y was made based on Abinawanto et al. (2016). The activator solution was prepared by diluting 0.263 g of NaCl; 0.037 g of KCl and 0.363 g of Tris-HCl with distilled aquabidest up to 100 mL. The solution was kept at 4 °*C* (Perchec et al., 1995). The 0.5% of eosin-Y solution was prepared by diluting 0.5 g of the eosin-Y with distilled aquabidest up to 100 mL.

### 2.5 Preparation of 0.15 M phosphate buffer and Giemsa solutions

The 0.15 M Phosphate buffer was made based on Abinawanto et al. (2016), whereas Giemsa solution was prepared according to WHO (2010). The 0.15 M Phosphate buffer solution was prepared by diluting 5.34 g of Na_2_HPO_4_.2H_2_O and 4.08 g of KH_2_PO_4_ with distilled aquabidest up to 200 mL. The solution was kept at 4 °*C* prior to use in the experiment (Abinawanto et al., 2016). The Giemsa solution was prepared by diluting one part of Giemsa stock solution with ten parts of 0.15 M Phosphate buffer, and was then filtered by Whatman paper no.1. The Giemsa solution was then kept at 4 °*C* (WHO, 2010).

### 2.6 Sperm collection

Four males weighing 800 ± 10.34 g were treated intramuscularly with Ovaprim (Syndel Laboratories Ltd. Nanaimo, Canada) at dosage of 0.8 mL kg-1 body weight. After 18 h, sperms were collected from individual male donors by a gentle abdominal stripping method (Muchlisin et al., 2010) and placed in 2 mL vials (Cryogenic storage vial, Nalgene Nunc International).

### 2.7 Sperm dilution

Fresh sperm was suspended in the diluent mixtures containing fish Ringer's solution, 10% methanol, and the respective egg yolk solution where applicable (Table 1). The composition of the solution was modified after Abinawanto et al. (2018). The dilution ratio of the fresh sperm and diluent solution was 1:10 based on Sunarma et al. (2007). The compositions of each component of the diluent solution and the ejaculated sperm are presented in Table 1. All treatment were subjected to the ejaculated sperm.

**Table 1:** The compositions of each component of the dilution solution and the ejaculated sperm.

Experimental Groups	10% Methanol (μL)	Fish Ringer (μL)	Egg yolk (μL)	Ejaculated sperm (μL)
0% Egg Yolk	50	450	0	50
5% Egg Yolk	50	425	25	50
10% Egg Yolk	50	400	50	50
15% Egg Yolk	50	375	75	50
20% Egg Yolk	50	350	100	50
25% Egg Yolk	50	325	125	50

### 2.8 Equilibration, freezing and thawing

Following Abinawanto and Putri (2017), the diluted sperm in 2 mL tubes was equilibrated at 4–5 °C in the refrigerator for 10 min then frozen at −10 °C in freezer for 48 h. After that, the frozen sperm was thawed at 40 °C for 1 min in a water bath (Abinawanto et al., 2013).

### 2.9 Sperm quality evaluation

The fresh sperm was evaluated for colour and pH. The preserved sperm was analysed for viability, and abnormality rates using a Boeco Trinocular Microscope (Boeco, Germany) equipped with a digital eyepiece camera (MDCE-5a). The microscope was connected to a computer equipped with an image driving software (Scopephoto 2.0.4).

### 2.10 Fish egg collection and fertilization

The eggs were collected from the mature female fish by gentle abdominal pressure, and the eggs were put in the plastic basin and kept at 5 °*C*. A total of 100 eggs were taken randomly then fertilized with the treated sperm. The fertilized eggs were incubated in different a plastic basin A total of 0.2 mL of eggs were mixed with 0.6 mL of thawed sperm (1:3 v/v) and two drops of tap water, and then mixed with a soft feather and left in contact for 5 min. A Completely randomized design was applied as shown in Table 1. The ovulated eggs obtained were divided in to the each treatment. The fertilization rate was observed 2 h after incubation. The fertilized eggs were transparent, while the unfertilized were opaque. The fertilization rate was calculated using the following formula: fertilization rate (%) = fertilized eggs/total number of incubated eggs x 100 (Yustina et al., 2003).

### 2.11 Statistical analysis

The replication of each treatment group was conducted based on Frederer formula, (*t*-1) (*n*-1) ≥ 15 (McDonald, 2014). The percentage data were arcsine transformed prior to analysis (Muchlisin et al., 2004). The data of sperm viability, abnormality, and fertilization were analyzed using one-way ANOVA then followed by the Tuckey's test to determine the best treatment. The analysis was conducted using SPSS 14. (SPSS, Chicago, IL, USA). The qualitative data such as semen colour, volume, and pH were analysed descriptively.

## 3 Results

**Figure 1:**
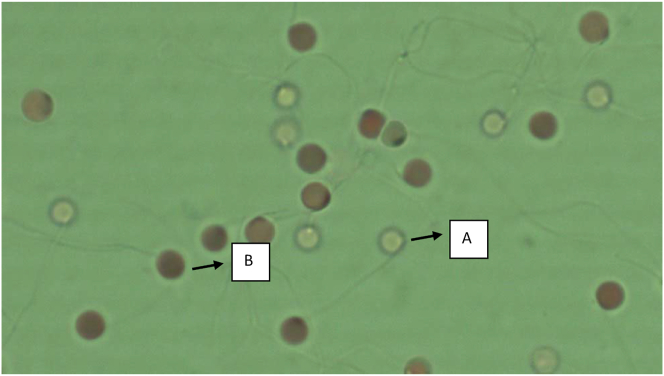
Viable Spermatoza (A) and Non-viable (B); The stain used was eosin-Y solution; Magnification 10 × 100.

**Figure 2:**
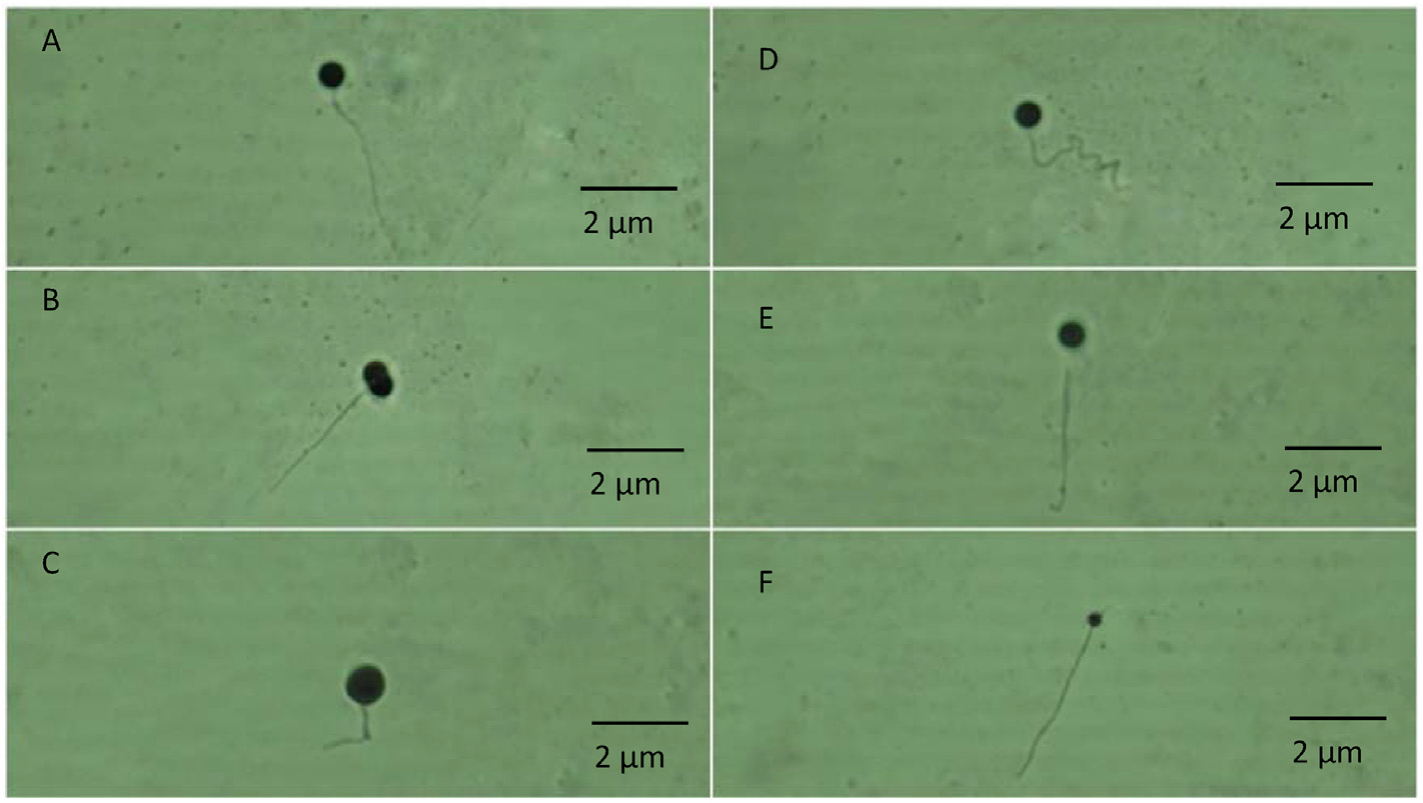
Normal (A) and Abnormal spermatozoa (B–F); Magnification 10 × 40. A. Normal spermatozoa; B. Double head spermatozoa; C. Macrocephallus spermatozoa; D. curved tail spermatozoa; E. Broken-off Tail spermatozoa; F. Microcephallus spermatozoa.

**Figure 3:**
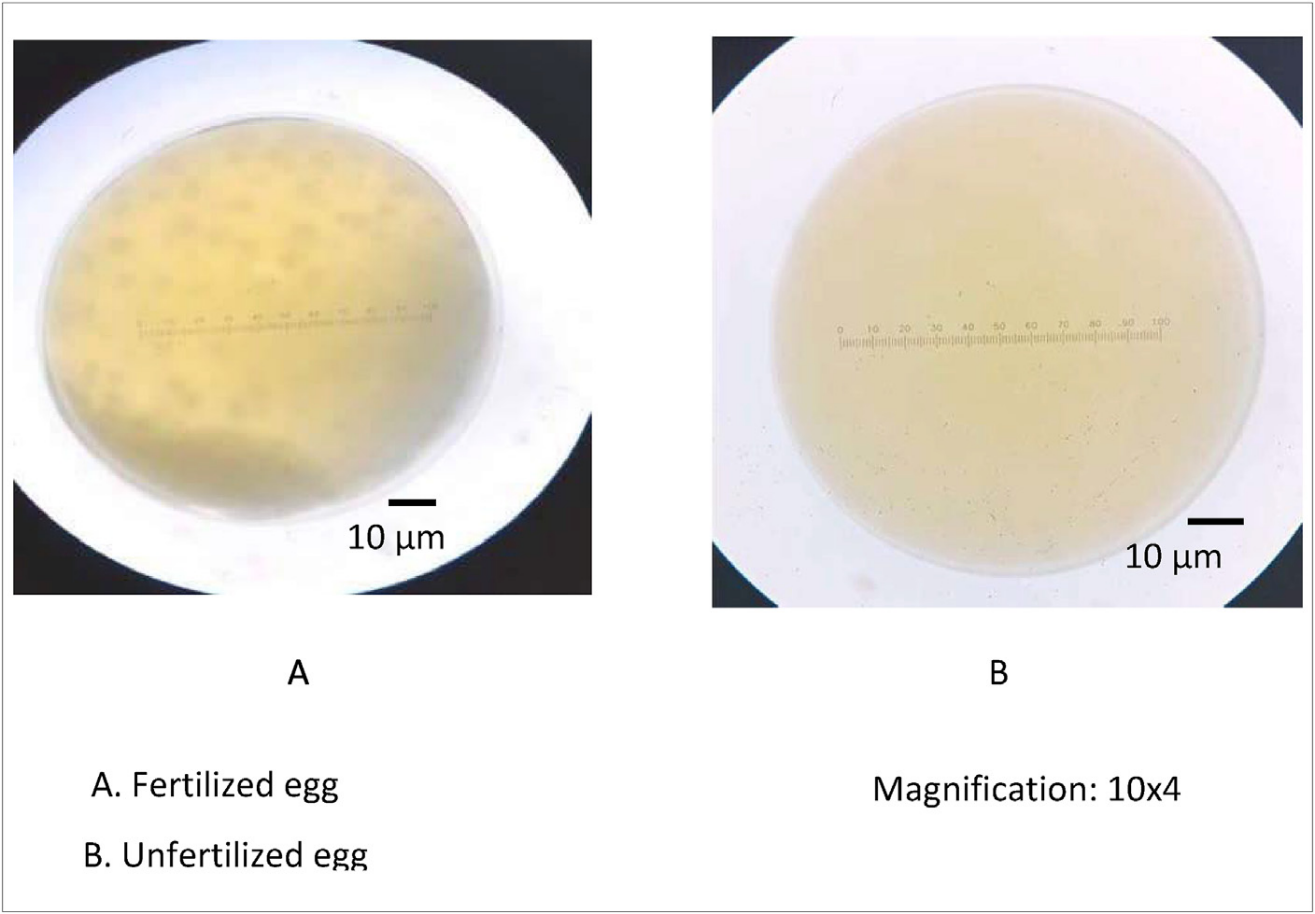
Fertilized egg (A) and Unfertilized egg (B).

**Figure 4:**
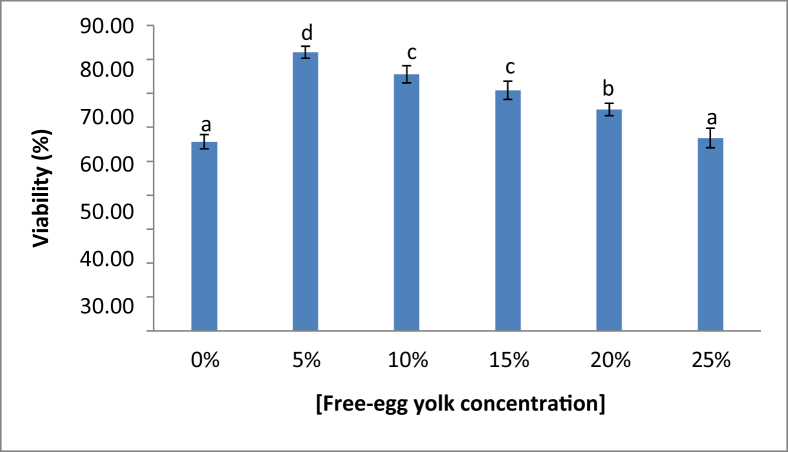
Spermatozoa viability rate. ∗) the different superscript demonstrated a significant different p<0.05 based on Tukey test.

**Figure 5:**
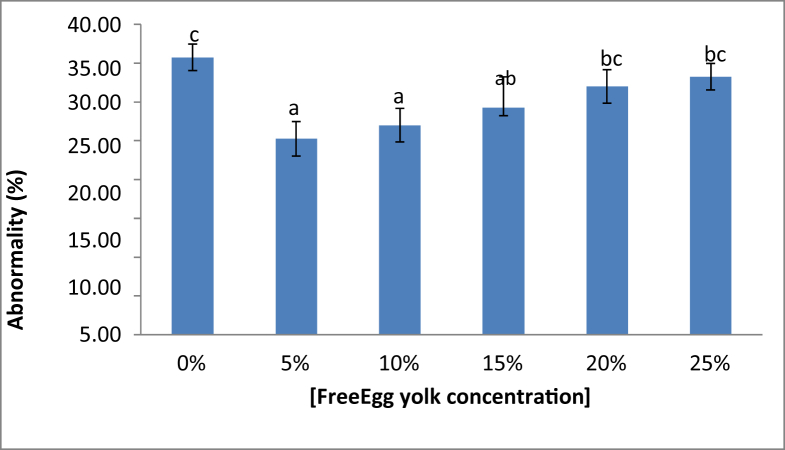
Spermatozoa abnormality rate. ∗)the different superscript demonstrated a significant (p<0.05) based Tukey test.

**Figure 6:**
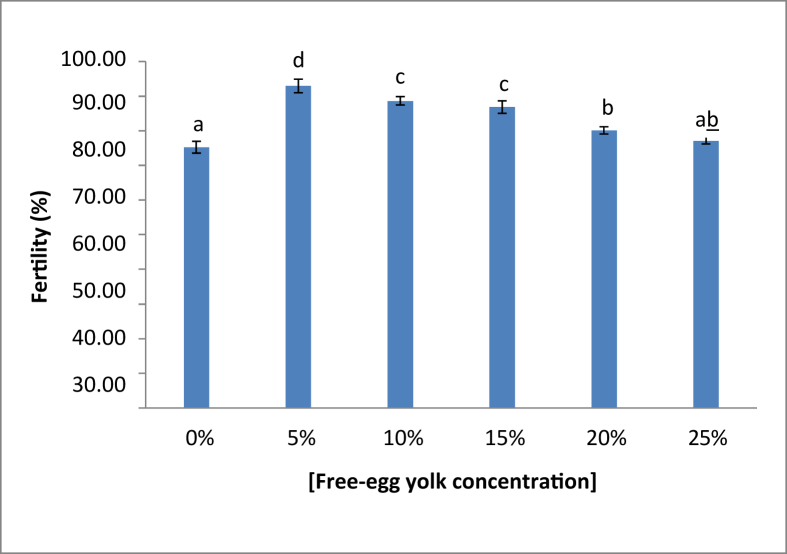
Fertility ratess. ∗)the different superscript demonstrated a significant (p < 0.05) based Tukey test.

The fresh sperm was milky white colour, volume was 1.5–2.5 mL, and pH was 8–8.5. Viable sperm showed a green colour on the sperm head (Figure 1a), whereas the non-viable sperm showed a pink or red colour on the sperm head (Figure 1b). A normal sperm, and the abnormal sperm were shown in Figure 2a, and in Figure 2b–f, respectively. The sperm abnormalities were classified based on Ax et al. (2008). Figure 3a shows the fertilized, whereas Figure 3b demonstrates the unfertilized egg. In general, the quality of fresh sperm was higher than cryopreserved sperm. The viability, abnormality, and fertilization rates of fresh sperm were 87.25 ± 1.71%, 20.75 ± 2.50%, and 95.10 ± 1.77%, respectively. However, the sperm quality has decreased gradually depending on the egg yolk solution concentration after 48 h preservation. The ANOVA test showed that the application of egg yolk solution in the diluents gave the significant effect on the sperm viability, abnormality and fertilization rates (*P* < 0.05). In general, the sperm quality was decreasing with increasing the concentration of egg yolk (Figure 4) in the extender solution. The use of 5% egg yolk gave the highest (*P < 0.*05) sperm viability (82.13 ± 1.75%). The lowest (*P < 0.*05) sperm abnormality (Figure 5) was also recorded at 5% egg yolk solution (25.25 ± 4.78%), and this value was lower (*P < 0.05*) than other treatments except the 10% egg yolk (27 ± 2.16%), and 15% egg yolk (29.25 ± 2.50%). In addition, the highest (*P < 0.05*) fertilization rate was recorded at the 5% egg yolk solution (92.96 ± 1.94%), and this value was higher (*P < 0.05*) than for egg yolk solution concentrations of 10%, 15%, 20%, 25%, and the control (Figure 6).

## 4 Discussion

The application of 5% egg yolk with 10% methanol in the fish Ringer's solution gives the best results on quality of God's fish spermatozoa 48 h post-thawing. The study revealed that sperm quality in the control (without egg yolk solution) was lower than the quality in the 5% egg yolk treatment. However, the quality of cryopreserved sperm decreased gradually at higher concentrations of egg yolks above 5%. This is might be due to the increase of the viscosity of diluent along with the increase of egg yolk concentration (Sakri, 2015), and preventing methanol entering the cell which reducing the protective effect of this intracellular cryoprotectant inside the cell, as egg yolk is known as natural extracellular cryoprotectant. In general, the natural cryoprotectants are non toxic, inexpensive, and environmentally friendly (Muchlisin, 2005; Muchlisin et al., 2015). Therefore, utilization of natural cryoprotectants as alternatives is highly recommended; however, it must be applied at the optimum concentration as observed in this study.

The sperm viability rate in the best treatment (10% methanol +5% egg yolk) of this study is higher than the combinations of cryoprotectants published in other studies; e.g.combinations of 20% skim milk +5% methanol for 81.75% viability rate (Abinawanto et al., 2016), and 0.7% honey solution +10% DMSO for 74.83% viability rate (Abinawanto et al., 2017). Therefore, we assume that the combination of methanol and egg yolk at concentration of 10% and 5% is a very effective cryoprotectant to maintain sperm quality of God's fish during cryopreservation. The simultaneous application of both intracellular and extracellular cryoprotectants resulted in better cryoprotective effect because these cryoprotectants gave complementary effect outside and inside of the cells (Akcay et al., 2004). Although using the same combination of the materials, after cryopreservation, we found that the viability rate of the sperm of God's fish was lower than tiger botia (Abinawanto et al., 2018) and Java barb (Abinawanto et al., 2013). In this study, the viable spermatozoa stained by eosin staining appeared transparent, because they got good membrane integrity. Therefore, the eosin stain colour could not penetrate inside the cell. On the other hand, the non-viable cells appeared pink (red) colour, because their membrane integrity had “broken”, so, the eosin staining diffused into the cell. Another method can be used to detect membrane integrity is flow cytometry, a widely applied technique for analysis of cell suspensions including sperm samples, and it has been used for assessment of sperm quality by analysis of plasma membrane integrity (Yang et al., 2016). Further, the sperm abnormality rate in the best combination (10% methanol +5% egg yolk) of this study was lower than the combinations of 20% skim milk +5% methanol, 26.25% abnormality in Abinawanto et al. (2016), and 0.7% honey solution +10% DMSO, 28.25% abnormality in Abinawanto et al. (2017). The highest motility rate (84.06 ± 1.67%) was shown by the combination of 5% egg yolk of free-range chicken and 10% methanol (Vardini et al., 2020). This result was higher than reported by Abinawanto et al. (2011) in goramy sperm which only 68.58%, and in kancra sperm (76.7%) which demonstrated by Junior et al. (2005). In contrast, the motility rate in this study was lower than in tiger botia sperm (96.43 ± 1.49%) compared to the previous report (Abinawanto et al., 2018). Indeed, the fertilization rate in this present study was also higher than tiger botia (Abinawanto et al., 2018), zebrafish (Rebocho, 2018), African catfish (Muchlisin et al., 2015), sharkminnow (Putra et al., 2017; Sunarma et al., 2007), bagrid catfish (Muchlisin et al., 2004), common carp (Akcay et al., 2004), and burbot, *Lota lota* (Linnaeus, 1758, Lahnsteiner et al., 2002). The presence of egg yolk in extenders incorporating 10% methanol provided additional protection to salmonid sperm during freezing and thawing (Jodun et al., 2006).

## 5 Conclusion

It is concluded that the combination of 5% of egg yolk of free-range chicken and 10% methanol are highly effective as a natural cryoprotectant agent for *Neolissochilus soroides* sperm storage at -10 °C for 48 h.

## Declarations

### Author contribution statement

Abinawanto Abinawanto: Conceived and designed the experiments, Analyzed and interpreted data, Contributed reagents, materials, analysis tools or data; Wrote the paper.

Nia Vardini: Performed the experiments.

Anang Hari Kristanto, Anom Bowolaksono: Analyzed and interpreted the data; Contributed reagents, materials, analysis tools or data.

Retno Lestari: Analyzed and interpreted the data.

### Funding statement

This work was supported by 10.13039/501100006378Universitas Indonesia (NKB-0015/UN2.R3.1/HKP.05.00/2019).

### Data availability statement

Data will be made available on request.

### Declaration of interests statement

The authors declare no conflict of interest.

### Additional information

No additional information is available for this paper.
